# Improving the Surface Quality of Network Microstructure Titanium Matrix Composites Using Electrochemical Milling Following EDM

**DOI:** 10.3390/ma18245628

**Published:** 2025-12-15

**Authors:** Yizhou Hu, Leheng Zhang, Sirui Gong, Zhenlong Wang

**Affiliations:** 1 School of Mechatronics Engineering, Harbin Institute of Technology, Harbin 150001, China; 2 Key Laboratory of Micro-Systems and Micro-Structures Manufacturing of the Ministry of Education, Harbin Institute of Technology, Harbin 150001, China

**Keywords:** titanium matrix composites, microstructure, electrochemical micromilling, electrical discharge machining, surface quality, parameter optimization

## Abstract

**Highlights:**

**What are the main findings?**

- ECM after EDM reduces Sa from 0.90 μm to 0.45 μm on NMTMCs.
- NaCl enables more uniform dissolution of NMTMCs than NaNO_3_.
- Standalone ECM causes selective corrosion and cannot finish NMTMCs.

**What are the implications of the main findings?**

- ECM is an effective post-EDM finishing method for NMTMCs.
- The combined EDM–ECM process improves surface integrity for precision use.
- The method offers a pathway for machining heterogeneous titanium composites.

**Abstract:**

Network microstructure titanium matrix composites (NMTMCs) possess excellent performance and are promising for aerospace applications, yet their microstructural heterogeneity poses substantial challenges to achieving high-quality micro-machined surfaces. The aim of this study is to evaluate electrochemical machining (ECM) as a post-processing method for improving the surface quality of NMTMCs after electrical discharge machining (EDM). This study systematically examines the effects of electrolyte concentration, machining voltage, and pulse frequency on surface roughness. Electrochemical measurements in NaCl and NaNO_3_ revealed that standalone electrochemical machining causes severe selective corrosion due to the large dissolution rate mismatch between TiBw reinforcements and the Ti-6Al-4V matrix, making it unsuitable for direct finishing. Accordingly, ECM was applied to EDM-prepared surfaces, and under optimized conditions (10 wt.% NaCl, 4.5 V, 200 kHz), ECM effectively mitigates the protrusions at the edges of discharge pits caused by the EDM process. Surface roughness (Sa) is significantly reduced from 0.90 μm to 0.45 μm, and the surface morphology becomes more uniform. These results demonstrate that ECM is a viable post-EDM finishing strategy for achieving high-quality micro-machining of NMTMCs.

## 1. Introduction

Titanium alloys are widely used in aerospace, aviation, automotive engineering, and other high-performance applications due to their excellent mechanical properties, corrosion resistance, and low density. In addition, their superior biocompatibility makes them highly suitable for biomedical applications such as artificial bone implants [1]. Further enhancement of their mechanical and functional performance can be achieved by incorporating reinforcing phases into the titanium matrix, leading to the development of titanium matrix composites (TMCs) [2].

Among them, network microstructure titanium matrix composites (NMTMCs) consist of a titanium alloy matrix reinforced with network-distributed hard phases formed through specialized fabrication processes. This unique microstructure endows the material with exceptional specific strength and high-temperature performance, making it a promising candidate for next-generation aero-engine components [3]. However, the high toughness of the titanium matrix, coupled with the non-uniform distribution and brittle nature of the reinforcement phases, results in extremely poor machinability, which significantly hinders its practical engineering application. Recent studies by Dong et al. on ultrasonic grinding have shown that the reinforcement fibers, when subjected to substantial mechanical stress, not only induce machining instability such as chatter but also tend to generate surface defects and residual stresses on the machined surface [4].

Recent studies have shown that other types of titanium matrix composites (TMCs) also exhibit severe machining difficulties. In addition to the intense tool vibration and wear commonly observed during cutting [5], various levels of surface quality degradation are frequently reported. Chen et al. [6] noted that, during the grinding of SiC-fiber-reinforced TC17 (SiC_f_/TC17), the complex fracture behavior of SiC fibers adversely affects the machined surface [7], resulting in defects such as microcracks and micropores [8]. Wang et al. [9], in their ultrasonic cutting study, emphasized that the stress field distribution near the SiC_f_/Ti interface plays a crucial role in the formation of surface damage. Xu et al. [10] explored laser-assisted grinding and found that softening of the matrix under thermal effects can significantly improve surface integrity. Marousi et al. [11] investigated the evolution of machined surfaces during the cutting of TMCs with PVD-coated carbide tools under progressive tool wear.

In contrast, electrochemical machining (ECM), as a non-contact material removal method, has been increasingly applied to the processing of various metal matrix composites in recent years. Niu et al. [12] reported that during electrochemical milling–grinding of TMCs, unavoidable stray corrosion significantly deteriorates the flatness of the machined surface. Using jet electrochemical micro-milling on (TiB + TiC)/Ti-6Al-4V, the same group further found that stray corrosion tends to occur along groove edges, thereby reducing dimensional accuracy [13]. Ma et al. [14] investigated in detail the influence of reinforcement particles on electrochemical corrosion behavior and subsequently applied electrochemical turning to TMCs, revealing that a rectangular cathode with lateral electrolyte outlets effectively reduces the final surface roughness. Nair et al. [15] described the material removal mechanism of SiC_p_/Al during ECM and observed surface pits of varying sizes and morphologies. Liu et al. [16] conducted electrochemical jet machining on SiC_p_/Al and analyzed the potential mechanisms behind the abnormal removal profiles. Miao et al. [17], working on the same material with electrochemical milling-grinding, noted that the tool path exerts a significant influence on process stability. Overall, ECM offers unique advantages for processing metal matrix composites; however, surface quality issues caused by stray corrosion and non-uniform dissolution still remain prevalent in many applications [18].

Electrical discharge machining (EDM) exhibits unique advantages in the micromachining of Ti-6Al-4V alloys [19,20] and various metal matrix composites (MMCs) [21]. However, the inherent complexity of the EDM process often leads to surface-related issues of varying severity [22]. To address these limitations, hybrid machining processes that integrate EDM with ECM have gained increasing attention, and numerous studies have demonstrated their potential to achieve more controllable surface quality. Nawaz et al. [23] significantly improved the machining accuracy of microstructures on quartz glass using micro-electrochemical discharge machining (ECDM). Ahmed et al. [24] incorporated selected electrolytes into the deionized-water dielectric used in conventional EDM, enabling simultaneous spark erosion and electrochemical dissolution, thus altering the material removal mechanism and facilitating the formation of desirable surface morphologies. Meng et al. [25] applied an abrasive-assisted synchronous EDM–ECM technique to titanium alloys and achieved noticeable improvements in surface roughness. Sharma et al. [26] performed micro-hole machining on Inconel 718 using a similar hybrid approach, and the resulting surface finish surpassed that obtained by standalone EDM or ECM. Dong et al. [27] developed a novel oil-in-water nanoemulsion possessing both insulating and conductive properties to enable EDM–ECM hybrid deposition machining, yielding high-quality surfaces. Singh et al. [28] achieved precise control of surface microstructures on Ti-6Al-4V by employing an in situ EDM–ECM hybrid method. Van et al. [29] explored a combined wire-EDM and wire-ECM approach within a single dielectric medium, achieving good surface quality and dimensional accuracy without compromising productivity. Sharma et al. [30] further investigated the mechanisms responsible for the improvement in surface roughness. Wu et al. [31] introduced a rotary EDM–ECM hybrid machining process for SiC/Al composites, enabling thinning and refinement of the EDM recast layer. Chen et al. [32] also developed a hybrid process combining surface protection with low-voltage assistance between high-voltage pulses, achieving exceptionally smooth surfaces at deep-hole entrances.

In summary, existing research on the machining of NMTMCs remains limited, and studies on their electrochemical processing are virtually absent. Moreover, investigations on similar materials indicate that conventional mechanical machining often results in surface quality issues to varying degrees. Therefore, this study first conducts electrochemical tests on NMTMCs to examine their characteristics and clarify the removal mechanisms of different constituent phases. Subsequently, electrochemical machining is applied to EDM-prepared surfaces, and the influence of machining parameters on surface quality is systematically explored. Through parameter optimization, a high-quality EDM–ECM hybrid-machined surface is ultimately achieved.

## 2. Materials and Methods

This study employs a combination of experimental techniques, including ECM, EDM, electrochemical testing, and surface characterization, to investigate the surface processing behavior of NMTMCs. All experiments were conducted using the machining and characterization facilities of the micro-manufacturing laboratory at Harbin Institute of Technology (China). An orthogonal experimental design and subsequent single-factor experiments were applied to analyze the influence of key ECM parameters on surface quality. The experiments were carried out using a three-axis micro-EDM platform integrated with a custom ECM power module, while the microstructure and surface morphology were analyzed using field emission scanning electron microscopy (SU8010, Hitachi High-Technologies Corporation, Hitachi, Japan) and a white-light interferometer (Zygo NewView™ 7300, Zygo Corporation, Middlefield, CT, USA).

The material used in this study is an NMTMC developed by Huang et al. [33]. The composite consists of a Ti-6Al-4V alloy matrix reinforced with TiB whiskers (TiBw) at a volume fraction of 5% (5 vol.%). In the Ti–6Al–4V alloy designation, the numbers “6” and “4” denote the mass fractions of Al and V (6 wt.% and 4 wt.%, respectively). The chemical composition is listed in Table 1. To expose the reinforcement phase clearly for microstructural observation, the surface of the NMTMC workpieces is pretreated prior to machining. The pretreatment involved sequential grinding with sandpapers of grit sizes ranging from P200 to P2000, followed by mechanical polishing using Cr_2_O_3_ abrasive paste with an average particle size of 1.5 μm.

**Figure 1:**
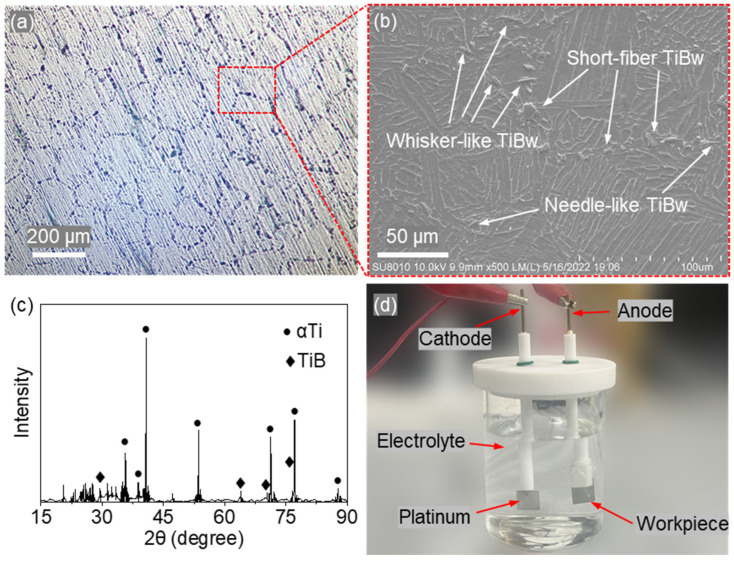
Microstructural characteristics and electrochemical test of NMTMCs: (**a**) Optical micrograph of the polished surface, (**b**) SEM micrograph showing TiBw reinforcements, (**c**) XRD pattern, (**d**) Schematic of the electrochemical test.

Figure 1a shows the surface morphology captured by an optical microscope (CNOPTEC MIT500, Chongqing Optec Instrument Co., Ltd., Chongqing, China). The reinforcement phase forms an interlaced network of strip-like regions, dividing the metallic matrix into irregular polygons with characteristic sizes of approximately 180–220 μm. A magnified view (Figure 1b) obtained by FESEM (SU8010, Hitachi High-Technologies Corporation, Hitachi, Japan) reveals that the reinforcement consists of needle-like, whisker-like, and short-fiber TiBw, densely aligned along the strip-shaped reinforcement regions. Phase analysis was performed using X-ray diffraction (XRD, X’PERT POWDER, PANalytical B.V., Almelo, The Netherlands) with a scanning range of 15–90°. As shown in Figure 1c, the material is composed primarily of Ti and TiB phases.

Samples for electrochemical testing were prepared using a DK7740P wire electrical discharge machining (WEDM) machine (Suzhou Sanguang Science &Technology Co., Ltd., Suzhou, China), operated under the discharge parameters listed in Table 2. The resulting specimens, with dimensions of 10 mm × 6 mm × 2 mm, were divided into two groups. The first group was left untreated, while the second group was ground, polished, and rinsed with deionized water prior to testing.

Electrochemical measurements were carried out using an electrochemical workstation (CHI660E, C241320c, CH Instruments Ins., Austin, TX, USA) equipped with a three-electrode cell. Sodium nitrate (NaNO_3_) and sodium chloride (NaCl) electrolytes—both commonly employed in the study of the electrochemical behavior and machining characteristics of Ti-6Al-4V alloys—were used as corrosion media, each with a mass fraction of 20%.

Tafel polarization tests were performed in both electrolytes using a three-electrode configuration, with a platinum electrode as the counter electrode and a saturated calomel electrode (SCE) as the reference. The anodic polarization curves were obtained under potentiostatic control with a scan rate of 1 mV/s and a potential range of −0.55 to −0.45 V. The electrolyte temperature was maintained at 30 ± 1 °C throughout the experiments.

For comparison, identical tests were conducted on Ti-6Al-4V alloy specimens under the same conditions, as listed in Table 1. Additionally, an electrolysis by-product analysis was performed in the setup shown in Figure 1d. The electrolysis process was carried out at a current of 3 A for 10 min, after which the by-products in the solution were filtered, dried, and collected for XRD analysis.

**Figure 2:**
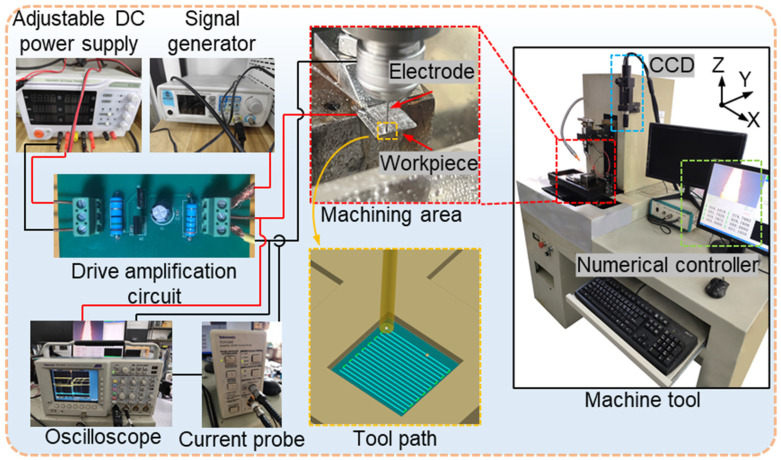
Experimental setup for the machining process.

As shown in Figure 2, all machining experiments were conducted on a three-axis micro-EDM system developed at Harbin Institute of Technology (China). The system consists of a transistor–RC composite pulse power supply, discharge monitoring system, servo feed mechanism, and a working-fluid circulation unit. It provides three feed directions (X, Y, and Z) with a positioning accuracy of up to 0.1 μm.

To enable ECM capability on this micro-EDM platform, our research group designed and integrated an external ECM power module, including a stabilized DC power supply (SS-3305D, Dongguan Bufan Electronics Co., Ltd., Dongguan, China), a custom drive amplification circuit, and a programmable signal generator (JDS6600, Hangzhou Junce Instrument Co., Ltd., Hangzhou, China). This integration enabled the original micro-EDM machine to perform ECM operations. During machining, a digital oscilloscope (Tektronix TDS 3034C, bandwidth 300 MHz, sampling rate 2.5 GS/s, Tektronix, Inc., Beaverton, OR, USA) was employed to record the voltage waveforms, while the current waveforms were measured using a current probe (Tektronix TCPA300, Tektronix, Inc., Beaverton, OR, USA) with a sensitivity of 1 A/V.

In the experiment, the plate-shaped NMTMC samples were first processed by EDM using a reciprocating tool path to produce square cavities with a side length of 20 mm and a depth of 0.1 mm. The EDM parameters are summarized in Table 2. Subsequently, the electrochemical milling process was performed along the same trajectory to refine the EDM surface. The surface topography and roughness of the processed samples were measured using a white-light interferometer (Zygo NewView™ 7300, Zygo Corporation, Middlefield, CT, USA) to compare the differences in their surface characteristics.

Since current density plays a decisive role in determining the material removal behavior and surface quality in ECM [15], and because it is strongly influenced by machining voltage [14,17] and electrolyte concentration [13], these two parameters are considered essential variables in ECM. In addition, the pulse frequency of the power supply affects the discharge period and ion transport behavior within the inter-electrode gap, thereby influencing the stability of the electrochemical reactions and ultimately the machined surface quality. Therefore, for investigating the surface integrity of NMTMCs under micro-scale ECM conditions, electrolyte concentration, machining voltage, and pulse frequency were selected as the key parameters for systematic study.

To investigate the effects of the three parameters on the final surface quality, an orthogonal experiment based on an L_9_(3^3^) design was conducted, as listed in Table 3. In this orthogonal experiment, three machining parameters—electrolyte concentration, machining voltage, and pulse frequency—were selected as independent factors, each at three levels. These factors are independently controlled in the experimental setup: electrolyte concentration is adjusted by solution preparation, machining voltage is regulated by the ECM power supply, and pulse frequency is controlled by the signal generator. Therefore, the three factors can be varied independently without mutual influence.

Based on the orthogonal experimental results, the optimal combination of parameters was identified and used as the baseline condition. Single-factor experiments were then conducted by varying one parameter at a time, while the remaining two parameters were fixed at the corresponding optimal levels determined from the orthogonal analysis. The specific fixed and varying parameter levels are summarized in Table 4. For each machining parameter condition, three independent samples were prepared, resulting in three independent repetitions per parameter condition. The final results represent the average of the three independent measurements to ensure reliability and reduce random experimental variation.

## 3. Results and Discussion

**Figure 3:**
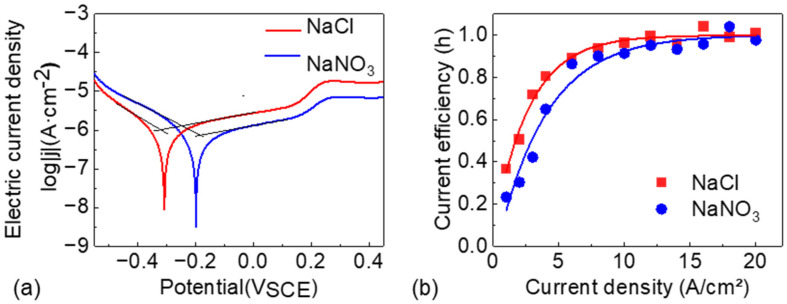
Comparison of electrochemical behavior in NaCl and NaNO_3_ solutions: (**a**) Polarization curves and (**b**) Current efficiency vs. current density in NaCl and NaNO_3_.

The Tafel polarization curves of NMTMCs in NaCl and NaNO_3_ electrolytes are presented in Figure 3a. From the curves, the corrosion potential (*E_corr_*), corrosion current density (*j_corr_*), and the cathodic and anodic Tafel slopes (*β_c_* and *β_a_*, respectively) are extracted, as summarized in Table 5. The corrosion potential of NMTMCs in the NaNO_3_ electrolyte is higher than that in the NaCl electrolyte, indicating a more positive corrosion tendency and thus better corrosion resistance in NaNO_3_. Moreover, the corrosion current density of NMTMCs in NaNO_3_ is slightly lower than that in NaCl. According to Faraday’s law, the corrosion rate is proportional to the corrosion current density, implying a slower corrosion rate in the NaNO_3_ electrolyte. This behavior can be attributed to the formation of a denser and more stable passive film in the NaNO_3_ solution, which suppresses material dissolution. In contrast, Cl^−^ ions in the NaCl electrolyte accelerate charge transfer and disrupt the passive film, making the material more susceptible to corrosion.

Figure 3b shows the current efficiency curves of NMTMCs under different current densities, with the experimental data fitted using an exponential decay function. The results indicate that the current efficiency in both electrolytes exhibits a similar trend: it increases sharply at low current densities, then gradually levels off, and eventually approaches a stable value close to 1.

At high current densities, the difference in current efficiency between the two electrolytes becomes negligible. However, a pronounced discrepancy is observed in the low-current-density region. For instance, when the current density is 6 A/cm^2^, the current efficiency in the NaCl electrolyte reaches approximately 90%, whereas in the NaNO_3_ electrolyte, a current density of around 10 A/cm^2^ is required to achieve a comparable efficiency. In micro-scale electrochemical machining, although high current density can enhance current efficiency, it typically degrades the surface quality. Consequently, low current density is preferred, under which the NaCl electrolyte demonstrates superior machining performance.

**Figure 4:**
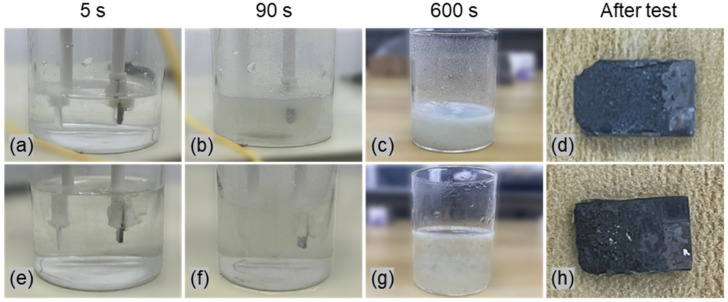
Comparison of NMTMCs in NaCl and NaNO_3_ electrolytes at different stages during electrochemical testing: (**a**–**d**) NaCl solution; (**e**–**h**) NaNO_3_ solution.

As shown in Figure 4, different electrolytes lead to distinct machining behaviors during electrolysis. At the beginning of electrochemical machining, a large number of bubbles are observed on the anode surface in both electrolytes, indicating that the bubbles are mainly generated by the electrochemical decomposition of water on the workpiece surface, as described by:

(1) $$4{\mathrm{OH}}^{-}\to {2H}_{2}O+O_{2}↑+4e^{-}$$

When the electrolysis time increased to 90 s, white precipitates appeared in both electrolytes, as shown in Figure 4b,f. With further extension of the corrosion time to 600 s, green precipitates are observed in both solutions, and the color is slightly deeper in the NaCl electrolyte (Figure 4c,g). Meanwhile, dense gas bubbles accumulate near the anode, and hydrogen ions in the electrolyte are reduced to hydrogen gas near the cathode. The reaction proceeds as:

(2) $${2H}^{+}+2e^{-}\to H_{2}↑$$

**Figure 5:**
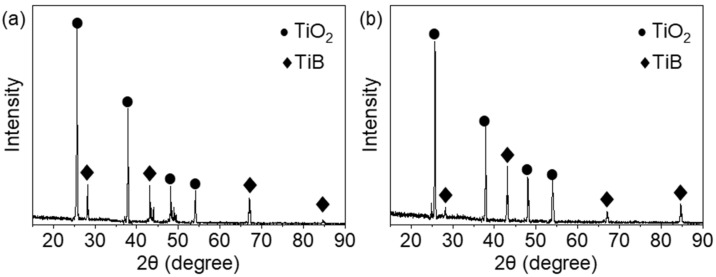
XRD patterns of the by-products obtained after electrochemical tests of NMTMCs in (**a**) NaCl and (**b**) NaNO_3_ electrolytes.

Figure 5 presents the XRD analysis of the electrolysis by-products formed in NaCl and NaNO_3_ electrolytes. Although the peak intensities differ slightly, the phase compositions are essentially identical, mainly consisting of TiO_2_ and TiB. Based on these results, the oxidation reaction of NMTMCs in NaNO_3_ electrolyte can be expressed as:

(3) $$\mathrm{Ti}+2H_{2}O-4e^{-}\to {\mathrm{TiO}}_{2}+4H^{+}$$

In contrast, Cl^−^ ions exhibit a strong activating effect on the titanium matrix, promoting corrosion through the formation of chloride complexes. The corresponding reactions are as follows:

(4) $$\mathrm{Ti}-4e^{-}+{4\mathrm{Cl}}^{-}\to {\mathrm{TiCl}}_{4}$$

(5) $${\mathrm{TiCl}}_{4}+2H_{2}O\to {\mathrm{TiO}}_{2}+4{\mathrm{Cl}}^{-}+4H^{+}$$

Therefore, the white flocculent precipitates observed in the electrolytes in Figure 4c,g can be identified as TiO_2_. In addition, under both electrolyte conditions, the B element in the by-products remains primarily in the form of TiB, with no new boron-containing phases detected. This indicates that the TiBw reinforcement in the NMTMCs does not undergo significant chemical reactions during electrochemical testing. The removal of TiBw is thus mainly attributed to the anodic oxidation and dissolution of the surrounding Ti-6Al-4V matrix, after which the unsupported TiBw detaches and is released into the electrolyte.

Furthermore, due to the composition of the Ti-6Al-4V matrix, aluminum and vanadium oxides are also formed as electrochemical by-products. However, their quantities are relatively low, resulting in weak diffraction signals that are difficult to distinguish in the XRD analysis.

The appearance of the samples after the completion of the electrochemical test is shown in Figure 4d,h. Owing to the inherent stochasticity of the electrochemical reaction process, the samples exhibit irregular overall shapes and surface morphologies. In addition, the pronounced heterogeneity in the constituent distribution within the NMTMCs results in noticeable variation across different regions of the surface after testing.

**Figure 6:**
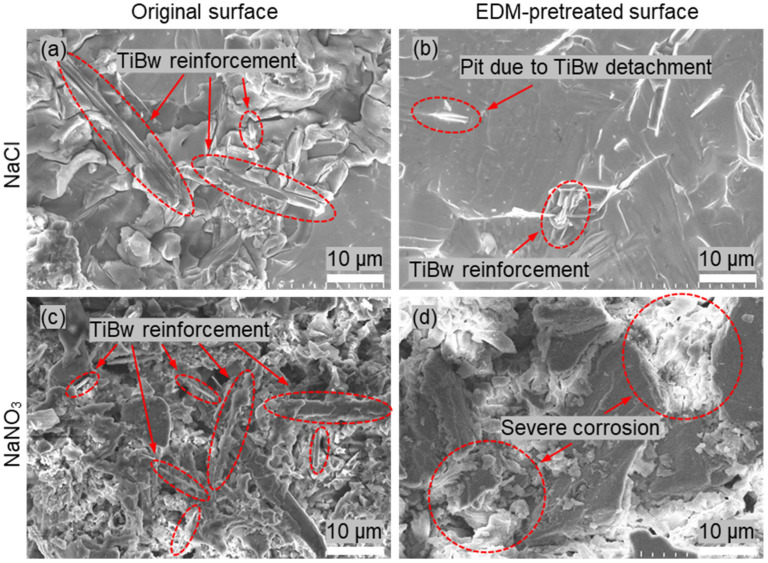
SEM images of NMTMCs after electrochemical tests in NaCl and NaNO_3_ electrolytes, showing the surface morphologies of original and EDM-pretreated samples. (**a**) Original surface in NaCl electrolyte; (**b**) EDM-preprocessed surface in NaCl electrolyte; (**c**) Original surface in NaNO_3_; electrolyte; (**d**) EDM-preprocessed surface in NaNO_3_; electrolyte.

Figure 6 presents the micro surface morphologies of NMTMCs after electrochemical testing in NaCl and NaNO_3_ electrolytes. As shown in Figure 6a,c, for the samples with the original unmachined surface, the presence of TiBw reinforcements and the significant difference in electrochemical activity between TiBw and the titanium matrix lead to markedly different dissolution rates during electrolysis. Consequently, the material removal becomes highly non-uniform.

In the NaCl electrolyte (Figure 6a), the titanium matrix is preferentially dissolved, while the TiBw reinforcements either remain on the surface or detach partially, resulting in a surface covered with numerous pits and protrusions. This produces a noticeably rough morphology and poor overall surface quality. In contrast, in the NaNO_3_ electrolyte (Figure 6c), the formation of a passive film on the titanium surface reduces the corrosion rate but further increases its non-uniformity. Large amounts of TiBw remain exposed on the surface, giving rise to a flocculent corrosion morphology with reduced flatness.

In comparison, as shown in Figure 6b, the surface morphology of NMTMC samples preprocessed by EDM is significantly improved. During EDM, the TiBw reinforcements are partially fractured or removed, resulting in a recast layer with a much lower TiBw content and a more homogeneous crystalline structure [34]. This structural uniformity allows the dissolution during electrochemical machining to become more consistent. As a result, in the NaCl electrolyte, a relatively flat surface can be obtained, with only occasional shallow pits or small amounts of residual reinforcements, leading to markedly better surface quality compared with the original samples.

However, when NaNO_3_ is used as the electrolyte (Figure 6d), the formation of passive films on the surface occurs in an uncontrolled manner, causing significant variations in the corrosion rate across different regions of the recast layer. This leads to severe stray corrosion, producing large corrosion pits and exfoliated areas. The surface integrity is consequently compromised, and the machining quality is notably degraded.

**Figure 7:**
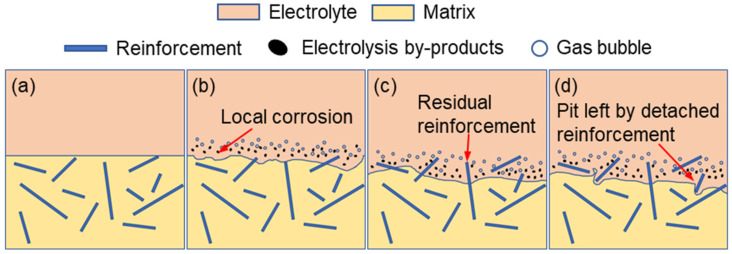
Schematic illustration of the surface evolution of the original NMTMCs during electrochemical testing. (**a**) Initial microstructure; (**b**) Local anodic dissolution; (**c**) Exposed reinforcements; (**d**) Pit formation after detachment.

Based on the above results, NaCl solution enables a relatively uniform material removal process for NMTMCs and provides higher current efficiency at low current densities, making it a suitable electrolyte for the electrochemical finishing of NMTMCs. Figure 7 summarizes the electrochemical corrosion mechanisms of both the original surface and the EDM recast-layer surface in NaCl electrolyte.

As illustrated in Figure 7a, TiBw reinforcements, which are essentially inert in the electrochemical reaction, are dispersed within the Ti-6Al-4V matrix. At the initial stage of electrolysis (Figure 7b), localized anodic dissolution of the Ti-6Al-4V matrix occurs on the workpiece surface, accompanied by the formation of electrolysis by-products and gas bubbles. As the matrix progressively dissolves, the embedded TiBw reinforcements gradually become exposed, as shown in Figure 7c.

With further processing, parts of the TiBw reinforcements lose their mechanical support due to the continued dissolution of the surrounding matrix and subsequently detach from the surface, leaving behind pits (Figure 7d). Consequently, for the original NMTMC’s surface, the inherent heterogeneity of the material and the selective dissolution between the reinforcements and the matrix inevitably lead to irregular morphologies and surface defects after electrochemical machining. This makes it difficult to achieve the high surface quality required for micro-scale precision machining applications.

**Figure 8:**
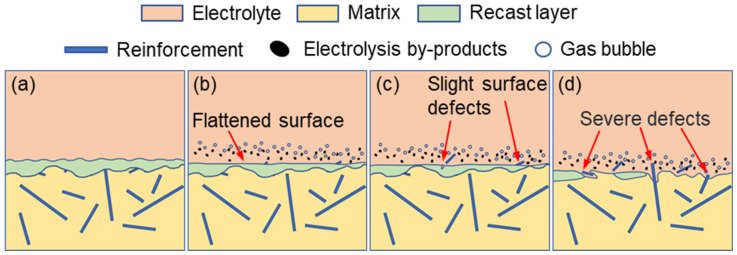
Schematic illustration of the surface evolution of EDM-pretreated NMTMCs during electrochemical testing. (**a**) Recast layer after EDM; (**b**) Uniform dissolution of recast layer; (**c**) Exposure or detachment of residual reinforcements; (**d**) Depletion of recast layer and transition to substrate corrosion.

In contrast, electrochemical finishing applied to the EDM-induced recast layer can significantly improve the machining quality of NMTMC surfaces. The corresponding corrosion mechanism is illustrated in Figure 8. After EDM, the recast layer exhibits slight surface undulations, and most TiBw reinforcements are fractured or removed during the process, while only a small portion remains embedded within the molten and solidified matrix, as shown in Figure 8a.

At the beginning of electrochemical machining, the surface of the recast layer undergoes uniform dissolution, resulting in a smoother and more refined morphology (Figure 8b). As machining proceeds, the few remaining reinforcements within the recast layer gradually become exposed or detach from the surface, producing minor localized defects, as shown in Figure 8c. With continued electrolysis, the recast layer becomes depleted in certain regions, exposing the underlying material, which subsequently undergoes the corrosion mechanism described in Figure 7. This transition leads to more pronounced and severe surface defects, particularly when portions of the recast layer still remain, as shown in Figure 8d.

Based on the above analysis, when electrochemical machining is performed on EDM-pretreated NMTMC surfaces in NaCl electrolyte, the material removal process becomes more stable due to the uniformity of the recast layer and the significantly reduced content of reinforcements. Consequently, a relatively smooth surface with improved quality can be obtained when the machining parameters and dissolution rate are properly controlled. Conversely, excessive electrolysis may deteriorate the surface quality.

**Figure 9:**
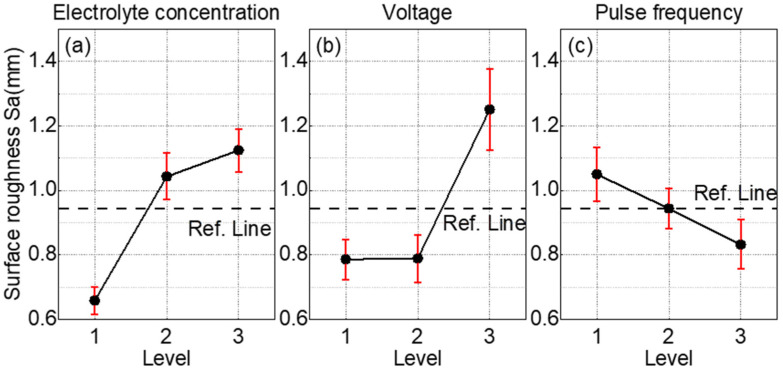
Main effects of machining parameters on the surface roughness of NMTMCs during micro electrochemical milling. (**a**) Effect of electrolyte concentration; (**b**) Effect of machining voltage; (**c**) Effect of pulse frequency.

Therefore, electrochemical milling can be applied as a post-processing technique to further refine the surfaces of EDM-milled NMTMCs and achieve superior surface quality. Figure 9 illustrates the main effects of electrolyte concentration, machining voltage, and pulse frequency on the surface roughness (Sa) obtained from the electrochemical micromilling of NMTMCs. The surface roughness parameter Sa was calculated in accordance with ISO 25178-2 [35], using areal surface texture measurements obtained from the white-light interferometer.

As shown in Figure 9a, the main effect of electrolyte concentration indicates that the surface roughness of NMTMCs increases with increasing NaCl concentration. This trend results from enhanced electrochemical reactions at higher concentrations. At low NaCl concentrations, the electrochemical reaction is relatively weak, and dissolution primarily occurs within the stage illustrated in Figure 9b,c. This behavior smooths the local undulations of the EDM-induced recast layer, thereby reducing surface roughness. As the concentration increases, stronger electrochemical reactions begin to penetrate the recast layer, causing stray corrosion and producing an uneven distribution of the residual layer. Consequently, the reinforcements become exposed or detached, forming pits and protrusions that markedly increase surface roughness. Once the recast layer is completely removed, the surface enters an over-dissolution state, further degrading surface quality and ultimately resulting in roughness levels higher than those of the original EDM surface.

As shown in Figure 9b, the main effect of machining voltage reveals that the surface roughness initially remains stable and then increases as voltage rises. At lower voltages, the electrochemical reaction energy is insufficient to penetrate the recast layer and only smooths its surface. With increasing voltage, the material removal depth exceeds the thickness of the recast layer, exposing the underlying reinforcements. Subsequent detachment of these reinforcements leads to pit formation and a deterioration of surface quality, thereby increasing roughness.

Figure 9c shows that surface roughness decreases with increasing pulse frequency. High-frequency pulses provide short reaction times followed by rapid interruptions, facilitating the removal of electrochemical by-products and maintaining a stable electrolyte condition in the machining gap. This reduces non-uniform dissolution and minimizes thermal effects during processing, improving surface quality. Moreover, higher pulse frequencies help regenerate the polarization layer, mitigating efficiency losses caused by polarization and promoting more uniform material removal, which reduces the influence of by-products on surface morphology.

The response table (Table 6) for means reveals clear differences in the influence of the three machining parameters on surface roughness. Electrolyte concentration shows the highest mean variation (Delta = 0.4658), indicating that it has the strongest effect on Sa. Machining voltage also contributes substantially (Delta = 0.4644) and is ranked second. In contrast, pulse frequency exhibits a much smaller mean variation (Delta = 0.2177), suggesting that its effect is comparatively weak. Accordingly, the parameters can be ranked in descending order of influence as follows: electrolyte concentration, machining voltage, and pulse frequency. The orthogonal experiment results indicate that an optimal combination for minimizing surface roughness consists of a 10 wt.% NaCl solution, a machining voltage of 4 V, and a pulse frequency of 200 kHz. Based on the optimal factor levels identified from the orthogonal analysis, single-factor experiments were subsequently performed to further investigate the detailed influence and variation trends of each machining parameter.

**Figure 10:**
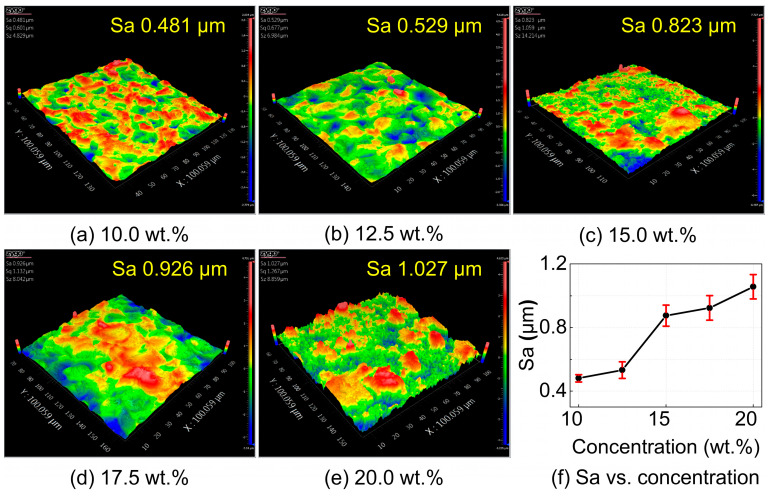
Surface morphology and roughness of NMTMCs after micro electrochemical milling at different electrolyte concentrations. (**a**) Surface morphology at 10 wt.%; (**b**) Surface morphology at 12.5 wt.%; (**c**) Surface morphology at 15 wt.%; (**d**) Surface morphology at 17.5 wt.%; (**e**) Surface morphology at 20 wt.%; (**f**) Surface roughness vs. electrolyte concentration.

The single-factor experimental results of surface roughness with respect to electrolyte concentration are shown in Figure 10. As the NaCl solution concentration increases, the surface roughness of the workpiece exhibits an upward trend. At low NaCl concentrations, the surface morphology of the workpiece is similar to that of the EDM-machined surface, but with a lower surface roughness, decreasing from 0.9 μm before electrochemical machining to 0.5 μm. As the NaCl concentration increases, stray corrosion causes the formation of larger pits on the workpiece surface. The surface morphology gradually loses the discharge pit stacking features from the EDM process and is replaced by irregular, chaotic undulations. With further increases in NaCl concentration, stray corrosion becomes more severe, leading to uneven corrosion on the workpiece surface. In some regions, the dissolution rate is slow, preserving the characteristics of the EDM surface; while in other regions, the dissolution rate is faster, resulting in irregular bottom depressions. Moreover, due to the exposure and detachment of the reinforcements, these areas contain many small pits and protrusions, leading to significantly degraded surface quality.

**Figure 11:**
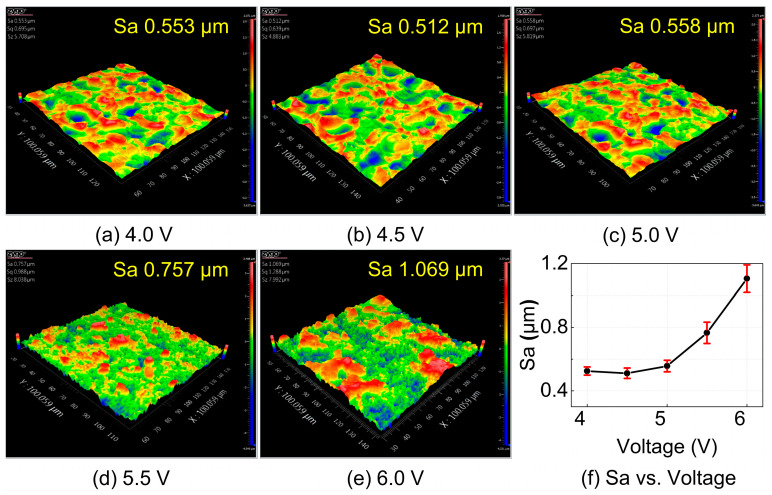
Surface morphology and roughness of NMTMCs after micro electrochemical milling at different voltages. (**a**) Surface morphology at 4.0 V; (**b**) Surface morphology at 4.5 V.%; (**c**) Surface morphology at 5.0 V; (**d**) Surface morphology at 5.5 V; (**e**) Surface morphology at 6.0 V; (**f**) Surface roughness vs. voltage.

The single-factor experimental results of surface roughness with respect to machining voltage, shown in Figure 11, indicate that surface roughness remains relatively stable between 4 and 5 V, but increases sharply between 5 and 6 V. Comparing the surface morphologies of the workpieces at 4 V and 5 V machining voltages, it is evident that at 4 V, the EDM-induced pits and protrusions are larger, whereas at 5 V, more micro-protrusions are observed. By examining the surface morphology at 4.5 V, it can be seen that due to the limitation of machining energy, at 4 V, the energy is insufficient, and the workpiece surface is not fully refined. At 5 V, the energy is excessive, leading to the complete removal of the recast layer in some areas and the exposure of reinforcements, resulting in poorer surface quality. Therefore, the optimal machining voltage for achieving the best surface quality is 4.5 V. With further increases in machining voltage, the excessive machining energy leads to over-corrosion of the workpiece, further degrading the surface quality.

**Figure 12:**
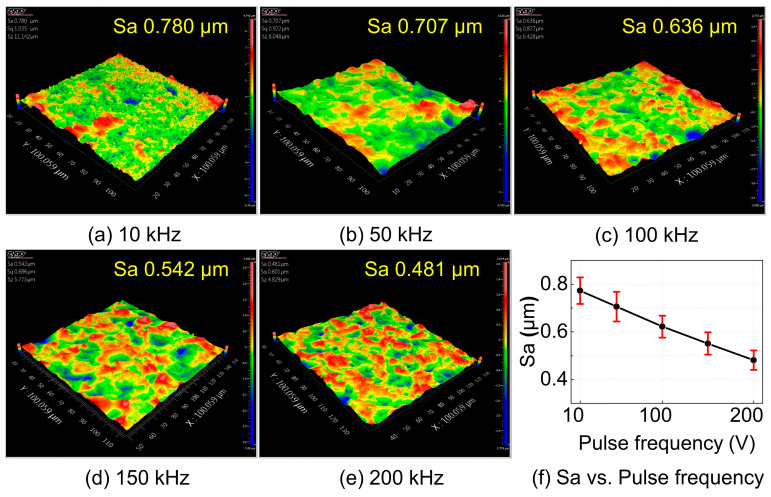
Surface morphology and roughness of NMTMCs after micro electrochemical milling at different pulse frequencies. (**a**) Surface morphology at 10 kHz; (**b**) Surface morphology at 50 kHz; (**c**) Surface morphology at 100 kHz; (**d**) Surface morphology at 150 kHz; (**e**) Surface morphology at 200 kHz; (**f**) Surface roughness vs. pulse frequency.

The single-factor experimental results of pulse frequency, shown in Figure 12, indicate that the surface roughness decreases as the pulse frequency increases. At lower pulse frequencies, the longer pulse-on time causes localized overheating on the workpiece surface. Due to the thermal conductivity mismatch between the reinforcements and the matrix, heat dissipation becomes non-uniform, which intensifies the uneven anodic dissolution. This leads to the formation of dense, serrated surface undulations and consequently deteriorates the surface quality. As the pulse frequency increases, the duration of each pulse is shortened, effectively suppressing excessive local heat accumulation. This alleviates localized overheating and promotes a more uniform dissolution process, thereby improving the overall surface quality.

By integrating the orthogonal experimental results shown in Figure 9 and Table 6 with the single-factor trends illustrated in Figure 10, Figure 11 and Figure 12, it can be concluded that electrolyte concentration and machining voltage exert the most significant influence on the surface quality of NMTMCs. This is mainly because these two parameters directly determine the current density in the machining zone, which governs the intensity and stability of the electrochemical dissolution process. As the electrolyte concentration and machining voltage increase, the surface roughness rises observably, and the recast layer formed during EDM is gradually exhausted. Consequently, the final surface roughness may even exceed that of the original EDMed surface. This phenomenon further verifies the validity of the surface removal mechanism model for NMTMCs proposed in Figure 7.

In contrast, when electrolyte concentration and machining voltage are maintained within reasonable ranges, pulse frequency mainly affects the machining stability by regulating the balance between electrochemical dissolution and reaction product removal. Since pulse frequency does not directly control the current density level, its effect on surface roughness is comparatively weaker and does not lead to a substantial increase caused by complete depletion of the recast layer.

**Figure 13:**
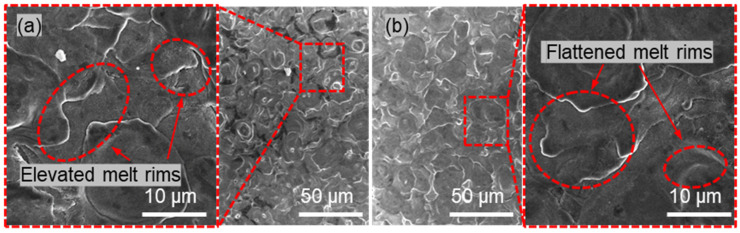
Evolution of surface morphology from EDM to electrochemical milling (**a**) EDM surface (**b**) Electrochemical milling surface.

Based on the results of the single-factor experiments, the electrochemical machining parameters are further optimized. A NaCl electrolyte concentration of 10 wt.%, a machining voltage of 4.5 V, and a pulse frequency of 200 kHz are selected to perform electrochemical micromilling on the EDM-machined NMTMCs surface. The resulting surface morphologies are compared in Figure 13. As shown in Figure 13b, the surface refined by electrochemical micromilling under the optimized parameters exhibits a significantly improved surface quality compared with the EDM-only surface shown in Figure 13a. The surface roughness is reduced from Sa 0.90 μm to Sa 0.45 μm.

Before electrochemical machining, the surface displays the characteristic stacked discharge pits produced by EDM, and the prominent protrusions around pit edges contribute to the relatively high roughness. After electrochemical machining, these protrusions are partially suppressed or removed, resulting in a smoother and more uniform surface morphology. Compared with standalone electrochemical machining, which often produces severe surface defects due to non-uniform corrosion, the combined method effectively avoids such issues while further improving the EDM surface quality.

Table 7 presents a comparison of the surface roughness obtained in this study with the results reported in recent ECM research on MMCs. Since electrochemical machining of NMTMCs is still in its early stages, the materials used in the comparison studies are not identical to those in this work. However, they all involve composite materials made of alloy matrices and hard reinforcements, which share certain similarities. Furthermore, although different researchers use either Sa or Ra values to characterize surface roughness, the difference between these two values mainly arises from the dimensionality of the measurements, thus allowing for some comparability.

As shown in the table, the surface roughness of SiC_p_/Al materials processed by ECM is relatively high, with values ranging from Sa 2.13 μm to Ra 1.51 μm. In contrast, the surface roughness of (TiB + TiC)/Ti6Al4V, a typical TMC, is lower, ranging from Sa 0.37 μm to Ra 1.48 μm. The surface roughness of Sa 0.45 μm obtained in this study falls within this range. It is noteworthy that, despite the challenges posed by the inhomogeneous internal composition of NMTMCs, the machining strategy proposed in this study successfully overcame these issues, enabling the production of surfaces that meet the stringent requirements for micro-scale and high-precision applications.

## 4. Conclusions

This work systematically evaluated the effectiveness of electrochemical micromilling (ECM) in improving the micro-machined surface quality of network microstructure titanium matrix composites (NMTMCs). The main conclusions are as follows:

1. Electrochemical behavior differences: NMTMCs exhibit distinct corrosion behaviors in NaCl and NaNO_3_. The activation effect of Cl^−^ in NaCl enables more uniform matrix dissolution and higher current efficiency, making it a more suitable machining medium.

2. Limitations of standalone electrochemical machining: The large electrochemical activity difference between TiBw reinforcements and the Ti-6Al-4V matrix leads to strong selective corrosion, preventing high-quality surfaces from being achieved by electrochemical machining alone.

3. Surface refinement and parameter effects of ECM: ECM on EDM-prepared surfaces effectively smooths micro-scale irregularities. Lower electrolyte concentration (10 wt.%), moderate voltage (4.5 V), and higher pulse frequency (200 kHz) suppress over-corrosion and improve dissolution uniformity, reducing surface roughness by about 50%.

4. Advantages of the combined process: EDM provides a homogeneous recast layer foundation, while electrochemical micromilling effectively eliminates surface irregularities. This combined process results in low roughness and high surface integrity, offering a reliable technological pathway for the precision micro-manufacturing of NMTMCs.

## Figures and Tables

**Table 1:** Chemical composition of NMTMCs and Ti-6Al-4V (wt.%). Reprinted from Ref. [34].

Material	Al	V	B	Fe	Si	O	C	N	H	Ti
NMTMCs	6.22	3.99	0.97	0.40	0.024	0.15	0.016	0.010	0.004	Bal.
Ti-6Al-4V	6.42	4.12	--	0.18	0.024	0.12	0.013	0.011	0.004	Bal.

**Table 2:** Machining parameters for EDM surface.

Experiment Parameters	Parameter Values
Electrode material	Tungsten
Electrode diameter (mm)	0.3
Capacitance (kpF)	10
Charging resistance (Ω)	1000
Discharge duration (µs)	100
Pulse interval (µs)	40
Polarity (workpiece)	Positive
Voltage (U/V)	100
Machining medium	Kerosene
Spindle speed (rpm)	2000

**Table 3:** Factor levels for the orthogonal experiment.

Factor/Level	NaCl Concentration (wt.%)	Machining Voltage (V)	Pulse Frequency (kHz)
1	10	4	10
2	15	5	100
3	20	6	200

**Table 4:** Machining parameters for electrochemical surface processing.

Experiment Parameters	Parameter Values
NaCl solution concentration (wt.%)	**10**/12.5/15/17.5/20
Machining voltage (V)	**4**/4.5/5/5.5/6
Pulse frequency (kHz)	10/50/100/150/**200**
Feeding speed (µm/s)	5.1
Machining gap (µm)	300
Electrolyte	NaCl Solution
Duty cycle	0.5

Bold values indicate fixed parameters corresponding to the optimal levels determined from the orthogonal experiments.

**Table 5:** Corrosion Parameters of NMTMCs Evaluated by Tafel slopes.

Electrolyte	*E_corr_* (mV_SCE_)	*j_corr_* (μA·cm^−2^)	*β_c_* (mV·dec^−1^)	*β_a_* (mV·dec^−1^)
NaCl	−308.15	1.047	155.53	270.52
NaNO_3_	−192.9	0.708	158.73	283.72

**Table 6:** Response Table for Means.

Factor/Level	NaCl Concentration	Machining Voltage	Pulse Frequency
1	0.6589	0.7866	1.0503
2	1.0432	0.7892	0.9438
3	1.1247	1.2510	0.8327
Delta	0.4658	0.4644	0.2177
Rank	1	2	3

**Table 7:** Comparison of surface roughness results for MMCs in recent electrochemical machining studies and the present work.

Workpiece Material	Machining Method	Surface Roughness/μm	Ref.
(TiB + TiC)/Ti6Al4V	Electrochemical Mill Grinding	Sa 0.37	[12]
(TiB + TiC)/Ti6Al4V	Jet Electrochemical Micromilling	Ra 1.03	[13]
(TiB + TiC)/Ti6Al4V	Electrochemical turning	Ra 1.48	[14]
SiC_p_/Al	Electro erosion milling	Sa 2.13	[15]
SiC_p_/Al	Electrochemical milling-grinding	Ra 1.51	[17]
NMTMCs	EDM+ electrochemical Micromilling	Sa 0.45	This work

## Data Availability

The original contributions presented in this study are included in the article. Further inquiries can be directed to the corresponding author.

## Abbreviations

Abbreviation	Meaning
MMCs	Metal matrix composites
NMTMCs	Network microstructure titanium matrix composites
TiBw	TiB whisker
ECM	Electrochemical micromilling
EDM	Electrical discharge machining
TMCs	Titanium matrix composites
FESEM	Field emission scanning electron microscopy
XRD	X-ray diffraction
WEDM	Wire electrical discharge machining
SCE	Saturated calomel electrode

## References

[1] Sharma S.K., Gajevic S., Sharma L.K., Pradhan R., Miladinovic S., Asonja A., Stojanovic B. (2024). Magnesium-titanium alloys: A promising solution for biodegradable biomedical implants. Materials.

[2] Krstić J., Jovanović J., Gajević S., Miladinović S., Vaxevanidis N., Kiss I., Stojanović B. (2024). Application of metal matrix nanocomposites in engineering. Adv. Eng. Lett..

[3] Gao N., Cui X., Luo J., Zhai X., Zhang T., Wang Z., Zhang Y., Ding H., Chen J., Geng L. (2024). Hot deformation behavior and processing map of novel quasi-network Ti_5_Si_3_/TiAl composites. Intermetallics.

[4] Dong G., Gao S., Wang L. (2022). Three dimensional shape model of TiBw mesh reinforced titanium matrix composites in rotary ultrasonic grinding. J. Manuf. Process..

[5] Wang M., Huan H., Zhao B., Ding W., Luo T., Yao R., Wu J. (2025). Evaluation of cutting performance of microtextured PCD tools on particle-reinforced titanium matrix composites. Int. J. Refract. Met. Hard Mater..

[6] Chen T., Xiao H., Feng S., Zhao B., Ding W., Qian N., Xu J., Wang Y. (2024). Heterogeneous components removal mechanism and grinding force model from energy aspect in ultrasonic grinding continuous fiber reinforced metal matrix composites. J. Mater. Process. Technol..

[7] Chen T., Feng S., Lin C., Ding W., Zhao B., Xu J. (2025). Force model based on heterogeneous components decoupling and machining behaviors of ultrasonic grinding continuous fiber-reinforced MMCs. Chin. J. Aeronaut..

[8] Chen T., Feng S., Lin C., Zhao B., Ding W., Xu J., Zhao Y., Zhu J. (2025). Unraveling the removal mechanisms of ultrasonic vibration-assisted grinding of continuous fiber-reinforced metal matrix composites: Experiment and simulation model. Front. Mech. Eng..

[9] Wang L., Gao X., Feng Q., Guo X., Li Z., An W., Xu W., Li Q., Yuan S. (2025). How does ultrasonic cutting affect the macroscopic deformation and microstructure evolution of fibre-reinforced titanium matrix composites?. Int. J. Mach. Tools Manuf..

[10] Xu D., Ai T., Shen Z., Ma S., Hossen M.S., Liao Z. (2025). Materials removal mechanism in laser-assisted grinding of SiC fibre-reinforced titanium alloy composite. Cirp Ann.-Manuf. Technol..

[11] Marousi M., Bejjani R., Balazinski M. (2025). Initial wear of cutting coated tools while machining TiMMC. Wear.

[12] Niu S., Huang K., Ming P., Qin G., Peng Y. (2024). Electrochemical mill grinding of (TiB + TiC)/Ti6Al4V composites using abrasive tool with bottom outlet holes. Micromachines.

[13] Niu S., Wang H., Ming P., Qin G., Ren L., Liu H., Li X. (2024). Electrochemical properties and jet electrochemical micromilling of (TiB + TiC)/Ti6Al4V composites in NaCl + NaNO_3_ mixed electrolyte. Materials.

[14] Ma X., Hu X., Cao X., Shen J., Lu Z., Li H. (2025). Optimizing electrochemical turning of titanium matrix composites: Enhancing efficiency with inclined cathode tools. Cirp J. Manuf. Sci. Technol..

[15] Nair A., Bizon W., Skoczypiec S., Uthayakumar M. (2024). Experimental investigations on electro erosion milling of Al-SiC metal-matrix composite. Mater. Manuf. Process..

[16] Liu W., Miao S., Gao M., Zhao Y., Wang Y., Zhao Y. (2025). Effect of dissolution behavior on electrochemical jet machining of SiC_p_/Al metal matrix composite. J. Mater. Process. Technol..

[17] Miao G., Fang X., Han Z., Qu N. (2025). Electrochemical milling-grinding of high volume fraction SiC_p_/Al composites with high surface quality via trajectory and process optimization. Cirp J. Manuf. Sci. Technol..

[18] Zhu Y.L., Liu G.D., Li Y., Tong H., Cao P.Y. (2024). A shunt-assisted silicon electrode for micro electrochemical machining. J. Micro Nano-Manuf..

[19] Jaiswal S., Kuriachen B., Mathew J. (2023). Experimental investigation into the evolution of microstructure and nanomechanical characterization of electric discharge deposited surface layers on Ti64. Measurement.

[20] Kuriachen B. (2024). Influence of surface texture of electric discharge machined Ti6Al4V on the surface wettability. Precis. Eng.-J. Int. Soc. Precis. Eng. Nanotechnol..

[21] Yao Z.Q., Ge J., Cao P.Y., Wu M., Qian J., Li Y., Reynaerts D. (2025). Advancements in process monitoring and quality control for electrical discharge machining: A comprehensive review. J. Mater. Process. Technol..

[22] Yue X.M., Fan J., Li Q., Yang X.D., Xu Z.K., Chen Z.Y. (2022). Influence of discharge gap on material removal and melt pool movement in EDM discharge process. Int. J. Adv. Manuf. Technol..

[23] Nawaz S.A., Cao P.Y., Tong H., Li Y. (2023). Micro ECDM scanning process with feedback control of flexible contact force. J. Manuf. Process..

[24] Ahmed S., Speidel A., Murray J.W., Ahmed N., Cuttell M., Clare A.T. (2022). Electrolytic-dielectrics: A route to zero recast electrical discharge machining. Int. J. Mach. Tools Manuf..

[25] Meng J.B., Wang S.K., Guan Q.Y., Dong X.J., Li H.M., Li L., Zhao G.Y., Zhao Y.G. (2022). Experimental investigation on simultaneous machining of EDM and ECM of Ti6Al4V with different abrasive materials and particle sizes. Int. J. Adv. Manuf. Technol..

[26] Sharma S., Dvivedi A. (2023). Simultaneous electrochemical and electrodischarge machining process: An approach to sustainable manufacturing. J. Manuf. Process..

[27] Dong H., Gong W.X., Qiu Y., Zhou J.P. (2024). Electrical discharge and electrochemical hybrid sinking machining using water-in-oil nanoemulsion. Int. J. Adv. Manuf. Technol..

[28] Singh R., Tiwari T., Rakurty C.S., Dvivedi A., Kumar P. (2024). Fluoride-ion enhanced the electrochemical surface texturing of Ti6Al4V alloy via in situ EDM and ECM with patterned tool. Int. J. Adv. Manuf. Technol..

[29] Van Riel T., Qian J., Lauwers B. (2024). Wire EDM roughing and Wire ECM finishing of 316L stainless steel on a single platform—An investigation of the combined strategy on surface quality and precision. Cirp Ann.-Manuf. Technol..

[30] Sharma S., Dvivedi A. (2025). Theoretical and experimental investigation of sustainable simultaneous electrochemical and electrodischarge machining process: A step towards green manufacturing. J. Electrochem. Soc..

[31] Wu X., Liu Z., Li Y., Yang H. (2025). Processing of silicon carbide particulate reinforced aluminum alloy composites using rotary electrochemical discharge machining. Mater. Manuf. Process..

[32] Chen H., Yang X., Yue X. (2025). EDM ECM composite processes of high-quality small hole with large aspect ratio using surface protection and low-voltage assistance. Int. J. Adv. Manuf. Technol..

[33] Huang L.J., Geng L., Li A.B., Yang F.Y., Peng H.X. (2009). In situ TiBw/Ti-6Al-4V composites with novel reinforcement architecture fabricated by reaction hot pressing. Scr. Mater..

[34] Zhang L., Hu Y., Gong S., Wang Z. (2024). Experimental Study on the Comparison between network microstructure titanium matrix composites and Ti6Al4V on EDM milling. Materials.

[35] ISO (2021). Geometrical Product Specifications (GPS)—Surface Texture: Areal—Part 2: Terms, Definitions and Surface Texture Parameters.

